# “You find yourself in a very humiliating situation”: experiences of people living with post-tuberculosis lung disease in Brazil

**DOI:** 10.3389/fpubh.2024.1431881

**Published:** 2024-12-24

**Authors:** Carlos Podalirio Borges de Almeida, Jennifer Joan Furin, Alberto Sumiya, Denise Rossato Silva, Carole Diane Mitnick

**Affiliations:** ^1^Faculty of Public Health, Federal University of Sul e Sudeste do Pará, Marabá, Pará, Brazil; ^2^Department of Global Health and Social Medicine, Harvard Medical School, Boston, MA, United States; ^3^Division of Infectious Diseases and HIV Medicine, Case Western Reserve University and University Hospitals Cleveland Medical Center, Cleveland, OH, United States; ^4^Santa Catarina Federal University (UFSC), Medical School, Curitibanos, Santa Catarina, Brazil; ^5^Faculdade de Medicina, Universidade Federal do Rio Grande do Sul (UFRGS), Porto Alegre, Brazil

**Keywords:** tuberculosis, chronic disease, post-tuberculosis lung disease, quality of life, health services accessibility, Brazil

## Abstract

**Background:**

Brazil remains one of the 30 countries with the highest tuberculosis (TB) and TB-HIV coinfection burden. Post-TB lung disease (PTLD) is a set of sequelae that can occur in people who have been cured of TB.

**Aim:**

To learn about the experiences of people living with PTLD (PLPTLD) and how healthcare workers (HCW) manage PTLD.

**Methods:**

An exploratory qualitative study with a purposive sample of PLPTLD and HCW from two different cities. Open-ended interviews were conducted using a semi-structured interview guide, which were recorded and transcribed. Two researchers analyzed the interviews using an inductive approach and applied a content analysis framework to define categories.

**Results:**

Forty-six participants were interviewed, and four categories emerged: PTLD as a social disease; stigma; the fragility of access; and limitations. The categories encompassed two main aspects like PTLD in activities of daily living and emotions in everyday life, for instance, challenges with preparing meals, getting a job, barriers to set clinical appointments or getting social assistance, and stigma.

**Conclusion:**

This study reveals specific needs from patients and providers such as a lack of knowledge about PTLD, physical limitations, and stigmatization due to PTLD. It is crucial to address these issues in public health policies.

## Introduction

Annually, 82,000 individuals are diagnosed with tuberculosis (TB), and treatment results in a microbiological cure for 68,4% of those diagnosed each year (1). Multidrug-resistant tuberculosis (MDR-TB) occurs in 1,000 people in Brazil annually which leads to health problems even after treatment ending (2). Brazil remains among 30 countries with the highest TB and TB-HIV coinfection burden (3, 4).

The post-TB lung disease (PTLD) is a set of sequelae in people who have been cured of their TB (5). Existing data indicate that typical symptoms of PTLD occur in up to 50% after treatment (6–8): residual coughing, weakness, dyspnea, sputum production, difficulties in climbing stairs, managing everyday work activities (9–17), resulting in reduced quality of life, exercise tolerance and weight loss.

A recommended standard for evaluation and management of PTLD was recently published (10). It included six elements to be considered after TB treatment completion: evaluating the presence of PTLD; assessing the impact of the disease on quality of life; selecting individuals who could benefit from pulmonary rehabilitation; developing a tailored program for pulmonary rehabilitation; evaluating the effectiveness of pulmonary rehabilitation; and developing proper health education strategies. The publication also lists priority topics and designs for future studies of PTLD highlighting the importance of guidance for PTLD management and evaluation. In Brazil, in December 2023, guidelines for screening, monitoring, or treating PTLD were published for the first time (18). At present, there is a lack of knowledge about the needs for health and well-being of these individuals and services provided to this population are scarce. Previous studies have shown the risk factors for PTLD progressing, among which are various levels of poverty, advanced age, and repeated treatment for TB (19). Moreover, PTLD is a long-lasting disease of high-magnitude. In addition to individual morbidity, PTLD also poses health systems challenges, including unnecessary hospitalization and re-treatment for active TB (19). Given the large burden of PTLD and the growing awareness, it is critical to comprehend the experience of people living with PTLD in Brazil. Such knowledge is critical to develop interventions and improve health and living conditions in the Brazilian context. Furthermore, it possesses the potential to assist other countries grappling with similar challenges. To that end, the present study aimed to learn about the life experiences of people with PTLD.

## Methods

### Design and study sites

This is an exploratory qualitative study of PLPTLD in the cities of Porto Alegre (state of Rio Grande do Sul) and Palmas (state of Tocantins). Palmas is also the Tocantins state capital and has 313,349 inhabitants, and the highest percentage (87%) of new TB cases with TB/human immunodeficiency virus (HIV) co-infection which are undergoing antiretroviral therapy. TB and HIV infection incidence are approximately 15,7 and 18,8 by 100,000 inhabitants, respectively. Its gross domestic product (GDP) is $6,803 *per capita*, and Human Development Index (HDI) score is 0.788. Porto Alegre is the Rio Grande do Sul state capital and has 1,492,530 inhabitants. It has a high frequency of loss to follow-up from TB treatment (29,9%). In this city, TB and AIDS incidence is 70,7 and 47,2 by 100,000 inhabitants, respectively, GDP *per capita* is $10,716, and HDI score is 0.805. We chose these cities because they could offer a diversity of experiences of PLPTLD.

### Participants

We recruited a purposive sample of 31 PLPTLD, based on TB sequelae [changes in imaging tests (cavitation, bronchiectasis, pleural thickening, fibrosis, and pulmonary hypertension), impairment in the walking test, and/or spirometric changes; or symptoms of respiratory impairment: cough, dyspnea, wheezing, decreased exercise capacity, and/or chest pain]. They were referred to the study by health-care workers (HCW) from the health system in each city. We also recruited 15 HCWs providing care to PLPTLD. The characteristics collected from HCWs were: gender, age, job, and work location (primary care/hospital); and from PLPTLD: gender and age.

Among 46 participants, 5 people with PTLD were recruited from the primary healthcare (PHC) level in Palmas and 26 from the Clinics Hospital of Porto Alegre (HCPA) in Porto Alegre. We approached them as indicated by the TB coordinator in Palmas and by the Pulmonology Service coordinator at HCPA in Porto Alegre. Nine HCWs were recruited from PHC in Palmas and 6 HCWs were recruited from the HCPA in Porto Alegre. In Porto Alegre, professionals from the pulmonology service at HCPA referred PLPTLD (based on TB sequelae symptoms previously described) and professionals who met the research criteria. In Palmas, the recruitment took place in a similar way, with professionals being directed by the city’s TB coordination and these workers directed PLPTLD. In both cities, the contact with the participants was via telephone, the PI (CPBA) called each potential participant (up to three times at different days and times using all phone numbers provided). For those who accepted to participate, an appointment was set up to perform consent and conduct the interview on a date and location selected by the participant.

### Inclusion criteria

The PLPTLD – (i) 18 years old or older; (ii) willingness to provide Informed Consent; (iii) experiencing PTLD as determined by health providers. Providers – (i) 18 years old or older; (ii) willingness to provide Informed Consent; (iii) work as a healthcare professional providing care to PLPTLD.

### Data collection

The interviews were conducted in Portuguese by the PI or a research assistant. Both are native Portuguese speakers and are trained in qualitative techniques. Individual interviews were conducted in person in locations selected by the participants, including hospitals, health centers, or their homes. The researcher asked the participants to share the following types of experiences as they relate to their symptoms after TB treatment: contact with healthcare professionals; support received from family members, friends, and colleagues; contact with health agencies and institutions; and performance of every-day activities. Also, we explored life changes related to TB diagnosis, treatment, and completion of treatment. The coding phase started after we had conducted a third of the interviews. A theoretical saturation of data was used to stop performing interviews. After coding the 31–36th interviews no new themes emerged and no changes in the codebook were produced, then saturation was reached (20).

The interviews followed a guide with open-ended questions, developed through a review of specialized literature. The interview guide presented the key questions from the interview—which sought information about the experiences, emotions, and actions of participants—, as well as probing questions (Supplementary material 1). The researchers involved in the study were committed to maintaining data confidentiality and anonymizing material or data obtained.

### Data analysis

The voice recordings from the interviews were transcribed in Portuguese manually by CPBA and a research assistant. Only the selected quotes for the manuscript were translated into English. CPBA and AS analyzed the content using an inductive approach and the content analysis framework as proposed by Laurence Bardin. The technique entails three essential stages: preliminary material analysis, exploration/coding, and treatment of data (categorization) (21). The researchers conducted manual coding independently for all the transcripts and then met periodically to discuss and resolve any discrepancies in labeling or coding. Dedoose version 9.0 was used only to organize data coding and extraction. Besides, the Consolidated Criteria for Reporting Qualitative Research (COREQ) checklist was followed (22).

### Reflexivity

As part of our qualitative research, we recognize the importance of reflexivity. We acknowledge that, as some of us are healthcare providers, we may have biases that affect our interpretation of participant’s experiences. Our research team consisted of three white women (one Brazilian and two Americans) and two Brazilian white gay men. However, only men conducted the interviews and coding. We acknowledge that gender may have influenced the interview process, data analysis, and write-up of the results in various ways. Regarding gender and race, it is understood that relations of power are in place, especially in healthcare where authority has a negative perspective. However, gender might have influenced interpretations in an empathic way as patients showed eagerness for rights and better cultural and socioeconomic status. Connected to that, it is also worth mentioning that the male researchers work as lecturers developing studies in the public health field, so they are directly involved in discussions that inequities claim to fight. Hence, they tend to be more on the left wing. Although the backgrounds show privileges, the study was well documented, findings were reevaluated many times and can be perceived throughout the text with reflexive notes and participants´ voice engagement. Throughout the research project, we discussed these areas with one another.

### Ethical aspects

The project was approved by the Research Ethics Committee of the Federal University of Pará (6.073.455), Hospital de Clínicas de Porto Alegre (6.161.106), and Fundação Escola de Saúde Pública de Palmas (6.054.753). All subjects included in this study provided written Informed Consent before data collection.

## Results

A total of 46 individuals participated in the interviews. The age, gender, city, and job are presented in Table 1. The interviews lasted an average of 32 min. For the total of 59 PLPTLD referred by HCWs, no telephone numbers were available for them. For 70 patients, three attempts were made to contact them at different times and days, but there was no response. During the telephone calls or review of their health records, it was discovered that 19 patients had passed away, 7 patients refused to participate in the study due to personal, financial, or safety reasons, and one patient was unable to speak due to another medical condition.

**Table 1:** Demographics characteristics of study participants.

Characteristics	People with PTLD (*n* = 31)	HCW (*n* = 15)
Age		
Mean	56.6	36.2
Range	39–78	26–54
Gender (*n*, %)		
Female	15 (48.4)	13 (86.7)
Male	16 (51.6)	2 (13.3)
City		
Palmas	5 (16.2)	9 (60.0)
Porto Alegre	26 (83.8)	6 (40.0)
Occupation HCW (*n*, %)		
Physician	–	6 (40.0)
Nurse	–	5 (33.3)
Physiotherapist	–	1 (6.7)
Community health worker	–	3 (20.0)
People with PTLD (*n*, %)		
Housewife	7 (22.6)	–
Retired	10 (32.2)	–
Other	14 (45.2)	–

**Figure 1:**
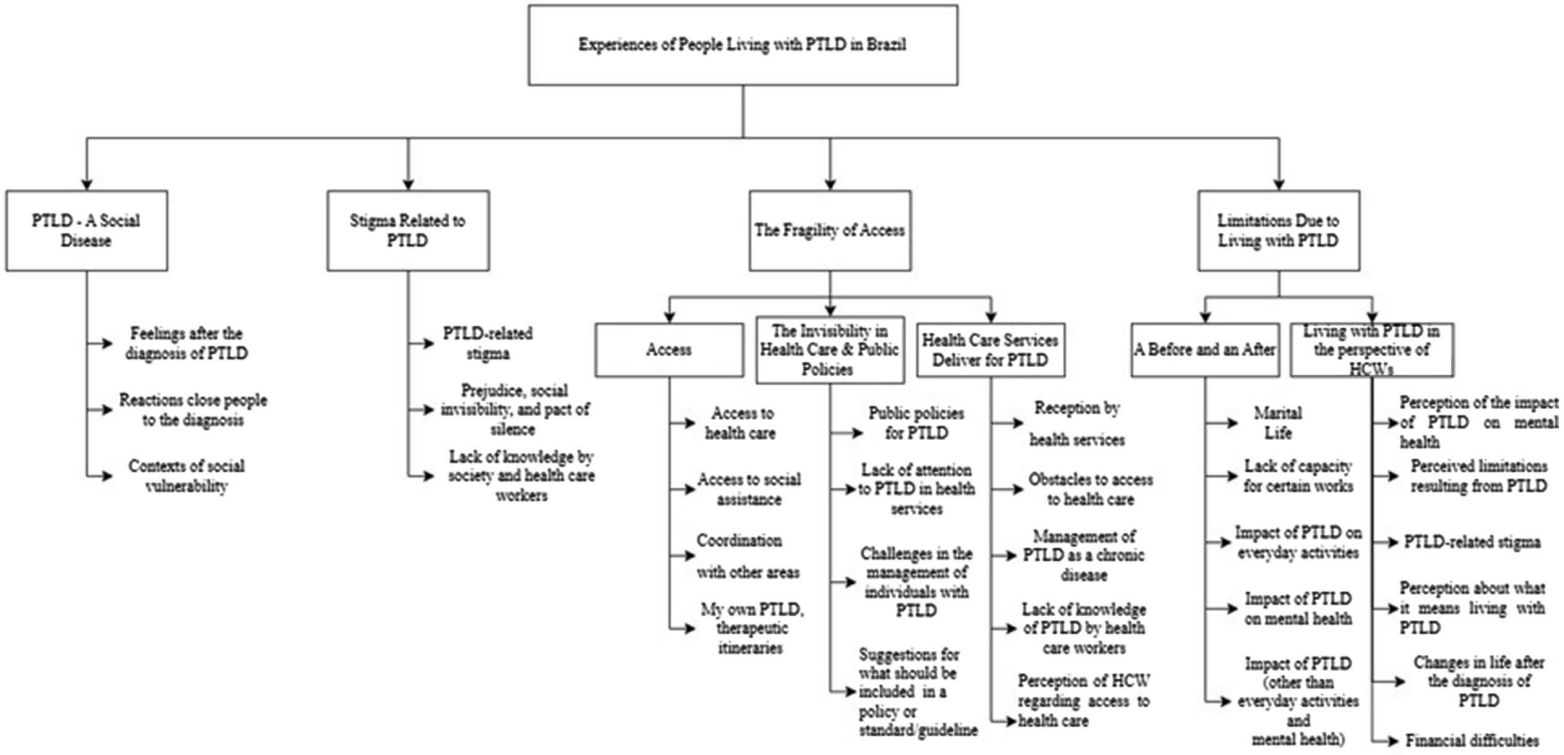
Coding tree for thematic analysis.

Figure 1 presents the categories and codes in the form of a coding tree chart. Five codes did not fall into any category: workload of healthcare workers, health work process, health education, patient knowledge about PTLD, and the impact of social vulnerability on how the patients deal with PTLD.

**Figure 2:**
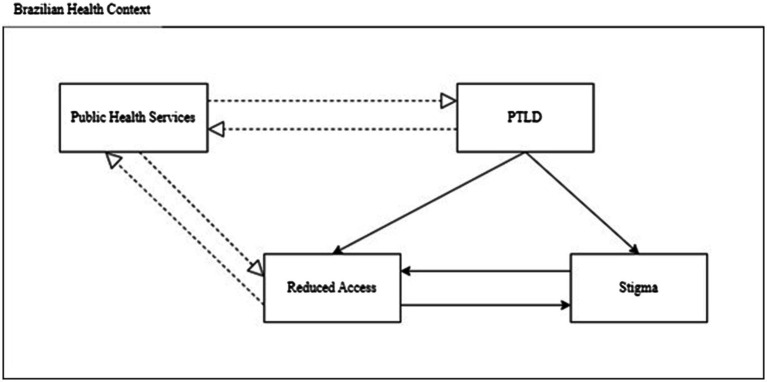
Structured relations among agents.

In order to better comprehend the dynamics of the main categories, Figure 2 was produced using Pierre Bourdieu’s notion of field, which encompasses structured relations among agents (PLPTLD and HCW). This framework reveals both historically established meanings and signs within these interactions, while also fostering a foundation for envisioning potential solutions. In this case, it is possible to visualize that PLPTLD and public health services are separated with poor direct connection (dashed lines). On the other hand, the perception of reduced access and stigma by PTLD have bidirectional strong relations affecting even more how services see them and provide care, despite governmental programs and previous professional training.

### Limitations due to living with PTLD

Most PLPTLD reported numerous obstacles to carrying out everyday activities. They adapted to mitigate PTLD effects. The impacts are felt by the PLPTLD both in ordinary tasks (such as preparing a meal, walking, and standing for some time) and in more strenuous ones (such as running). Many participants reported inability to perform household chores as well as to perform any type of work outside of their home. These limitations affect their mental health. PTLD changed their lives:

*“My life is limited to this. Everything is difficult: walking, sunbathing over there… Actually, when I sunbathe over there, I have to carry the oxygen cylinder with me. And there is no outlet there. The limitations are plenty, and the activities I can do are few.” (PLPTLD, male, age 74).*

*“No, it’s not possible, it’s a profession where you need to be standing. And you also need to climb a step, do a lot of things. It’s going to be hard and complicated. And my profession also involves dealing with the public. It requires moving a lot, holding people or running after them. No way, I cannot do this anymore, I cannot run anymore.” (PLPTLD, male, age 42).*

*“I mean, it’s impossible to feel good. I felt bad. Especially because I did not have support from anyone. I had nothing. I could not prepare a meal. I could not… I felt like garbage. Really incapable, you know?” (PLPTLD, female, age 39).*

Some respondents mentioned that the limitations worsened their emotional state, while others stated that it heightened the likelihood of developing mental disorders, leading them to seek treatment and support:

*“So, this left me battling depression for about 3 years. And then things started falling into place after I started going to church and seeing a psychiatrist. I managed to reverse the situation. And now I feel good. Of course, I get worried sometimes, mainly when I cannot see a specialist, it worries me.” (PLPTLD, female, age 58).*

Individuals reported difficulty in their sexual relations due to PTLD and because of that essential aspects of intimacy were compromised considering physical strength, respiratory capacity and the need for oxygen:

*“I worry, doctor, even about sex. Because whether you want it or not, my dear, what about the physical effort? Will you have the physical condition to satisfy your partner? Do you understand it? I admit it that this makes things very hard.” (PLPTLD, male, age 41).*

*“Even romantic relationships… I could not be in one anymore… Because I cannot have… any sort of sexual relation.” (PLPTLD, female, age 41).*

The HCW expanded their comments on the impacts of PTLD, which sometimes led to social isolation. Several dimensions were weakened by PTLD, including financial, social, and emotional. HCW also reported that PLPTLD brought moments of fear, stigma, and insecurity, especially soon after diagnosis. Lastly, the difficulties of physical functions are the most prevalent and visible, having devastating impacts.

*“Moving around. Exactly. Because everything is more complicated. You are not alone. You carry an oxygen cylinder, which is really heavy. You see what I mean? It is complicated to go about your normal day. It is hard to understand it until you see the patient. Then they remove the oxygen to talk to you. And in the middle of the talk, they cannot continue. And they put on the oxygen again. It is really hard to understand it.” (HCW, female, age 31).*

*“About the sequelae, the patient asks me ‘Will I ever return to work?’ And another patient tells he works on the farm doing manual labor. And he complains about shortness of breath. Right? Regarding any physical effort. And what will he do? Retire? I have a patient trying to do this. He says he needs to retire because he cannot work.” (HCW, female, age 49).*

### PTLD – a social disease

The expression of feelings and the sharing of experiences in statements of PLPTLD and of HCW unveil social matters related to PTLD. TB disease frequently manifests itself in contexts of social vulnerability. PTLD exacerbates other social aspects in the lives of the individuals. Several participants reported that reduced income, food insecurity, substance addiction, housing, and other factors seem to promote the emergence of PTLD and aggravate the disease. These circumstances hinder individuals’ ability to seek necessary care and engage in healthy behaviors. Besides, it seems because of their PTLD they face new or ongoing social stressors.

There is a bi-directional relationship between their vulnerability and the challenges to their health and well-being: poverty increases the risk of worsening TB/PTLD, while the emergence of PTLD increases poverty for them. For some, TB leads to them falling into poverty and now they cannot get out because of PTLD.

*“And then, at the end of the day, you want to take a shower and try to get something to eat. And because of my financial situation, I do not eat so well, you know? Because we have just the basic at home—rice and beans, if there is some bone-in meat I can prepare some broth. It happens because I have limitations, you see? I mean, I have no income, so I cannot afford it.” (PLPTLD, male, age 41).*

*“I cannot remember exactly how long it was, but he did not take his medication [medication to relieve PTLD symptoms] for about 3–4 months. He could not buy it, it was too expensive.” (HCW, female, age 49).*

### Stigma related to PTLD

The PTLD-related stigma arises in the statements of several PLPTLD and HCWs. They experienced it in multiple situations, including family environments, search for health care, church etc. However, for some participants, it appears that there is an unspoken agreement of silence within society regarding PTLD—people avoid discussing or learning about it. This is because PTLD symptoms such as persistent coughing, shortness of breath, or even carrying an oxygen cylinder can lead people to think that they are not cured of TB and that they can still transmit the microorganism. These stigmatizing experiences intensify the suffering and the social isolation among these individuals.

*“It’s about prejudice. Sometimes, it hurts more than the sequelae. Because, as I see it, it’s been so long, but when I say I had tuberculosis and talk about that, people look at me in an unpleasant way, do you know what I mean? As if someone who is by my side or who lives with me could still get the disease. That’s how I feel: the sequela that hurts the most is this one.” (PLPTLD, female, age 44).*

*“People feel… It’s hurtful for me to say it… They feel disgusted at us. Then you say you are… And they start to say: Do not be near him. Hey, do not get near him.” (PLPTLD, male, age 46).*

### The fragility of access

HCW and PLPTLD acknowledged that the health system is, to some extent, insufficient to solve their needs. However, they emphasized the importance of increasing access to healthcare services (lack of services/physical therapy, waiting times, appointment scheduling/limited hours, lack of staff etc.) and more coordinated services from different levels of care, but in this case, mainly with social assistance/insurance because of integral supports for the needs arising from PTLD, which are not only physical and encompass several dimensions of their lives.

*“And I’ve been saying all this time that I need a specialist. After I left Clínicas, I could not get one anywhere. Because… No one takes charge. So it’s been… It’s been a year I’ve been waiting for a spirometry. It’s been a year I’ve been waiting for a tomography. You see?” (PLPTLD, female, age 58).*

*“Oh, what they say to me? Let me remember what the last doctor said to me. That’s a big deal, right? Because you are not on sick leave by INSS [National Social Security Institute by the Brazilian government], and you also cannot work. If I were to depend on INSS, I’d be already dead. I never managed to get it.” (PLPTLD, female, age 58).*

*“That’s why we are going there, because she needs a medical report to apply for sick leave through INSS. And we are going to talk to social assistance too, since they are the ones who can help her. Because she is genuinely unable to work, you see? I think she is eligible for that disability benefit, something like that, I’m not sure, I know the government provides some kind of benefit like this. So we are going to check with social assistance about this.” (HCW, female, age 30).*

The HCWs reported experiences and perceptions that reveal neglectful care when it comes to individuals who had TB, developed PTLD, or have the potential for PTLD. On the other hand, it is also mentioned the lack of official guidelines regarding the management of PTLD as an obstacle to improving healthcare. Furthermore, they highlighted that some aspects like the assessment of PTLD after treatment completion, referral to physical therapy, and addressing mental health should be governmental policy standards for PTLD.

*“He sought assistance for more than one reason. And he did not say he received this diagnosis and that he requires medical monitoring. During screening of patients, depending on the health care worker, often times the patient does not say anything about their TB background and the worker also does not ask about it. This is a huge flaw.” (HCW, female, age 49).*

*“We do not look for sequelae of tuberculosis. We treat them [for TB]. When the patient notices they have some sort of sequela, they come back. If patients do not return, we do not do anything.” (HCW, female, age 26).*

*“It’s important to build a post-treatment, something that considers the person in a holistic manner, beyond the physical symptoms. There are mental health symptoms too and the need for social reintegration. This forms the entirety of the individual, and I think there’s no way to separate these things. When you consider a flow, if you think about protocols separately, you are not really promoting post-treatment holistically, you are just looking at one aspect of post-treatment. And that’s not truly seeing the individual as a whole. (HCW, female, age 29).*

In addition, HCWs stated that even if healthcare services receive PLPTLD well and professionals are willing to help them, access continues to be an obstacle. Some of the HCW-reported issues include poor health facilities accessibility, limited knowledge among HCWs about PTLD, a shortage of human resources, and difficulty in managing PTLD. Furthermore, HCWs pointed out that PTLD is or should be considered a chronic disease.

*“We should also see the social part of it because it is a long treatment. Maybe the person does not have food at home. This happens. So this patient needs help. We try to do this in several ways here. But it’s hard. And permanent. It’s a permanent need. Look, you have sequelae. And a higher risk of death.” (HCW, female, age 31).*

*“It’s not easy here at our unit because demand is very high. We have few workers in the therapy area. And sometimes the community we serve is very big and there are other diseases that also require monitoring. This is the problem. Sometimes this is the biggest difficulty.” (HCW, female, age 49).*

*“Look, I believe they should be treated as chronic patients. Because the link with them is lifelong, they demand constant help and will continue to show up.” (HCW, female, age 27).*

However, hospital HCWs perceived PLPTLD care was delivered in a more positive way.

*“I think they are very well received because we have several medical specialties. First, the patient sees a general practitioner, who usually requests an x-ray examination. Based on the results, the doctor identifies alterations and refers the patient to a pneumologist. So we are always monitoring these patients.” (HCW, female, age 29).*

*“And the patients, as any other person, notice when they are not welcome somewhere or when their rights are denied. For sure this creates some resistance for them to visit these places, right?” (HCW, female, age 29).*

## Discussion

The present study reveals a diverse set of challenges experienced by PLPTLD who need ongoing services. Reporting the experiences of PLPTLD and HCWs is an important step toward heightening awareness about obstacles faced by healthcare services and difficulties accessing structured and continued support. This study informs through qualitative research how PLPTLD and healthcare are intertwined in their experiences. As postulated by Lambert and Lambert (23), descriptive qualitative studies aim to summarize the daily events in the life of individuals or groups of individuals. Results can contribute to the understanding of the long-term impact of pulmonary infectious diseases, especially TB.

The PTLD results from a complex interplay between organism, host, and environmental factors. The most frequent TB sequelae include not only structural lung damage and infectious complications, but also psychosocial morbidities such as anxiety and/or depression, social isolation, persistent socio-economic impairment, and catastrophic costs (7).

People affected by PTLD have shortened life expectancy and increased risk of recurrent TB. In this way, patients after TB present with a wide range of consequences from completely asymptomatic to severe disability. Cross-sectional data from TB survivors suggest a high prevalence of chronic respiratory symptoms some years after treatment completion, including breathlessness and chronic cough, so these symptoms could lead to stigmatization (6). Approximately one-third of TB survivors face a considerable burden of morbidity, including reduced health-related quality of life (18). Furthermore, the mortality rate post-treatment is almost three times higher than the general population (24).

Besides PTLD, TB can cause several types of sequelae. In a recent clinical statement on post-TB health and wellbeing, the authors evaluated the psychosocial and socioeconomic effects in TB survivors. Poor HRQoL in PTLD patients is associated with impaired social life, economic losses, and persistent symptoms (25). The decrease in physical capacity resulting from chronic respiratory diseases impacts the economy and families’ livelihoods (26). People cured of TB may find themselves with long-term socio-psychological consequences and there are no current recommendations for mental health evaluation (6). One study (27) demonstrated that almost a third of total disability-adjusted life-years (DALYs) accruing 15 or more years after incident TB. A recent review on the social aspects of chronic respiratory diseases revealed that stigma, loneliness, and isolation can have a significant impact on the health outcomes of people suffering from these diseases. Also, the findings suggest that there is a pressing need for social efforts to address the lived experiences of stigma, which influences self-care (28), as dealing with body capacity also means living with vulnerability and uncertainty.

Another review on stigma experiences in people with chronic obstructive pulmonary disease (COPD) found stigma was frequently identified as a core component of participants’ lived experience, with detrimental effects on illness self-management and engagement with health and social care (29). Stigma negatively affects individuals and communities. It causes emotional distress, limited social interactions, and negatively impacts medication adherence and help-seeking. Healthcare providers and employers also contribute to stigma (29). PTLD might provoke anxiety as you cannot predict when and how symptoms will be worsened. These insecurities and fear produce many avoiding situations as mechanisms of protection and over precaution like visiting doctors constantly (30).

Negative impacts of stigma were found in other COPD studies, mostly during the interactions with HCWs and informal carers. Participants describe feeling undeserving of support: finding it difficult to share their concerns, and feeling their illness is underserved or not taken seriously by the healthcare system (31, 32), resulting in delays in seeking care (32). Respiratory patients usually feel themselves fragile and in doubt to endure life as it is, then hope starts to fade away gradually when apparently simple obstacles seem harder and harder to cope with, but effective rehabilitation can cause courage to go on (33).

Also, the existing stigma surrounding chronic respiratory diseases can be attributed to the perceived link between chronic coughing and infectiousness (26). This fact brings the perception of loss of freedom, sometimes being forced to stay home, reducing the number of activities and as a result starting to procrastinate, which reinforce bitterness, shame and resentfulness of PTLD. Dependency is another factor related to stigma because most of the time in patients’ discourse they strove not to dwell in self-pity by trying to see PTLD as a project for life (34).

Understanding health delivery service from a patient’s perspective, including factors influencing healthcare seeking behavior, is crucial when treating diseases. PLPTLD pathways may serve as indicators of the difficulties in obtaining adequate care. In this way, various factors are involved like economic barriers, integration of services, number of visits, cost of seeking care, level of education, and social and cultural context (35, 36).

For Endalamaw et al. (37), access to services affects satisfaction through lack of services, a long distance from health facilities, financial difficulties, individual acceptability of the services and waiting a long time to receive healthcare, might result in unmet healthcare needs. It includes perceived poor technical competence of health workers implying poor quality of care, so if TB patients perceive their care providers to be technically incompetent, it is understandable that the care they receive might be of poor quality.

In this sense, post-TB care is an essential part of comprehensive care according to TB HCWs in British Columbia, Canada. However, they believe that the lack of resources and time could hinder the implementation of such care (38). WHO has also recognized the need to provide interventions for people with TB-associated disabilities that affect their quality of life and social well-being. It is vital to incorporate the needs of this population in the rehabilitation, care, and TB programs (39). Physiotherapy programs have been found to be effective in improving symptoms associated with chronic respiratory diseases such as breathlessness, pain, immobility, and weight loss. Additionally, they improve social and intimate relationships (40). In this sense, a qualitative study on pulmonary rehabilitation for PLPTLD in Kyrgyzstan conducted with TB-HCW and PLPTLD found that participants were completely positive regarding the physiotherapy programs implementation in the country (41).

### Limitations

There are some limitations to this study that should be considered. Firstly, the study participants were a small sample of PLPTLD and HCW who were recruited from two cities in Brazil. Therefore, the findings may not be representative of the broader population of PLPTLD in Brazil or other regions.

## Conclusion

The study reveals specific needs from patients and providers such as a lack of knowledge about PTLD, physical limitations, and stigmatization due to PTLD. It is crucial to address these issues in public policies. Moreover, the results can help us to understand how PLPTLD accesses healthcare services and to propose improvements in this regard. The data enables the creation of programs for pulmonary rehabilitation specifically tailored to address the limitations faced by PLPTLD, including breathlessness and fatigue. Additionally, the data can serve as a tool to plan local coping strategies for PTLD, comprising public awareness campaigns and the creation of social support mechanisms for PLPTLD. Results from the present study could be hypothesis generating for future studies such as disease impact in the PLPTLD quality of life, and impoverishment due to PTLD. These results can also inform the development of more tailored care for people with PTLD in Brazil and elsewhere.

## Data Availability

The raw data supporting the conclusions of this article will be made available by the authors, without undue reservation.

## References

[1] Byrne AL Marais BJ Mitnick CD Lecca L Marks GB. Tuberculosis and chronic respiratory disease: a systematic review. Int J Infect Dis. (2015) 32:138–46. doi: 10.1016/j.ijid.2014.12.016 25809770

[2] Migliori GB Lunab JC Kurhasanic X Van den Boomd M Viscae D D’Ambrosio L. History of prevention, diagnosis, treatment and rehabilitation of pulmonary sequelae of tuberculosis. Presse Med. (2022) 51:104112. doi: 10.1016/j.lpm.2022.104112 35124102

[3] WHO. World Health Organization global tuberculosis report 2023. Geneva: WHO (2023).

[4] Ministério da Saúde. Secretaria de Vigilância em Saúde. Plano Nacional pelo Fim da Tuberculose como Problema de Saúde Pública. Brasília: Ministério da Saúde (2017).

[5] Daniels KJ Irusen E Pharaoh H Hanekom S. Post-tuberculosis health-related quality of life, lung function and exercise capacity in a cured pulmonary tuberculosis population in the Breede Valley District, South Africa. S Afr J Physiother. (2019) 75:1319. doi: 10.4102/sajp.v75i1.1319 31392293 PMC6676936

[6] Allwood BW Byrne A Meghji J Rachow A van der Zalm MM Schoch OD. Post-tuberculosis lung disease: clinical review of an under-recognised global challenge. Respiration. (2021) 5:1–13. 10.1159/000512531 33401266

[7] Visca D Centis R Munoz-Torrico M Pontali E. Post-tuberculosis sequelae: the need to look beyond treatment outcome. Int J Tuberc Lung Dis. (2020) 24:761–2. doi: 10.5588/ijtld.20.0488, PMID: 32912378

[8] Visca D Centis R Ambrosio LD Muñoz-Torrico M Chakaya ST. The need for pulmonary rehabilitation following tuberculosis treatment. Int J Tuberc Lung Dis. (2020) 24:720–2. doi: 10.5588/ijtld.20.0030, PMID: 32718406

[9] Shaw JA. Post-tuberculosis lung disease: exposing the elephant in the room. AJTCCM. (2021) 27:39. doi: 10.7196/AJTCCM.2021.v27i2.150, PMID: 34430864 PMC8327676

[10] Migliori GB Marx FM Ambrosino N Zampogna E Schaaf HS van der Zalm MM. Clinical standards for the assessment, management and rehabilitation of post-TB lung disease. Int J Tuberc Lung Dis. (2021) 25:797–813. doi: 10.5588/ijtld.21.0425, PMID: 34615577 PMC8504493

[11] Ranzani OT Rodrigues LC Bombarda S Minto CM Waldman EA Carvalho CRR. Long-term survival and cause-specific mortality of patients newly diagnosed with tuberculosis in Sao Paulo state, Brazil, 2010-15: a population-based, longitudinal study. Lancet Infect Dis. (2020) 20:123–32. doi: 10.1016/S1473-3099(19)30518-3, PMID: 31676242 PMC6928568

[12] Visca D Tiberi S Pontali E Spanevello A Migliori GB. Tuberculosis in the time of COVID-19: quality of life and digital innovation. Eur Respir J. (2020) 56:2001998. doi: 10.1183/13993003.01998-2020, PMID: 32513783 PMC7278505

[13] Migliori GB Tiberi S Zumla A Petersen E Chakaya JM Wejse C. MDR/XDR-TB management of patients and contacts: challenges facing the new decade. The 2020 clinical update by the global tuberculosis network. Int J Infect Dis. (2020) 92S:S15–25. doi: 10.1016/j.ijid.2020.01.042, PMID: 32032752

[14] Meghji J Gregorius S Madan J Chitimbe F Thomson R Rylance J. The long term effect of pulmonary tuberculosis on income and employment in a low income, urban setting. Thorax. (2020) 76:387–95. doi: 10.1136/thoraxjnl-2020-215338, PMID: 33443228 PMC7982936

[15] Schultink MP Kerstjens HAM Ter Beek L Zondag T Brijan R de Lange WCM. Assessment of TB treatment on patient well-being. Int J Tuberc Lung Dis. (2021) 25:315–7. doi: 10.5588/ijtld.21.0816 34160348

[16] Kawahara K Tabusadani M Yamane K Takao S Kuroyama Y Matsumura Y. Health-related quality of life associates with clinical parameters in patients with NTM pulmonary disease. Int J Tuberc Lung Dis. (2021) 25:299–304. doi: 10.5588/ijtld.20.0790, PMID: 33762074

[17] Ozoh OB Ojo OO Dania MG Dede SK Adegboyega OA Irurhe NK. Impact of post-tuberculosis lung disease on health-related quality of life in patients from two tertiary hospitals in Lagos, Nigeria. Afr J Thorac Crit Care Med. (2021) 27:47. doi: 10.7196/AJTCCM.2021.v27i2.135 PMC8327683 34430871

[18] Silva DR Santos AP Visca D Bombarda S Dalcolmo MMP Galvão T. Brazilian thoracic association recommendations for the management of post-tuberculosis lung disease. J Bras Pneumol. (2023) 49:e20230269. doi: 10.36416/1806-3756/e20230269 PMC10760438 38198346

[19] Mpagama SG Msaji KS Kaswaga O Zurba LJ Mbelele PM Allwood BW. The burden and determinants of post-TB lung disease. Int J Tuberc Lung Dis. (2021) 25:846–53. doi: 10.5588/ijtld.21.0278, PMID: 34615582 PMC8504494

[20] Guest G Bunce A Johnson L. How many interviews are enough? Field Methods. (2006) 18:59–82. doi: 10.1177/1525822X05279903

[21] Bardin L. Análise de conteúdo. Lisboa: Edições (1977).

[22] Tong A Sainsbury P Craig J. Consolidated criteria for reporting qualitative research (COREQ): a 32-item checklist for interviews and focus groups. Int J Qual Health Care. (2007) 19:349–57. doi: 10.1093/intqhc/mzm042, PMID: 17872937

[23] Lambert VA Lambert CE. Qualitative descriptive research: an acceptable design. Pacific Rim Int J Nurs Res. (2012) 16, 255–6.

[24] Silva DR Mello FCQ Migliori GB. Diagnosis and management of post-tuberculosis lung disease. J Bras Pneumol. (2023) 49:e20230055. doi: 10.36416/1806-3756/e20230055 37194818 PMC10171303

[25] Nightingale R Carlin F Meghji J McMullen K Evans D van der Zalm MM. Post-TB health and wellbeing. Int J Tuberc Lung Dis. (2023) 27:248–83. doi: 10.5588/ijtld.22.0514 37035971 PMC10094053

[26] Egere U Shayo E Chinouya M. “Honestly, this problem has affected me a lot”: a qualitative exploration of the lived experiences of people with chronic respiratory disease in Sudan and Tanzania. BMC Public Health. (2023) 23:485. doi: 10.1186/s12889-023-15368-6, PMID: 36915117 PMC10010645

[27] Menzies NA Quaife M Allwood BW Byrne AL Coussens AK Harries AD. Lifetime burden of disease due to incident tuberculosis: a global reappraisal including post-tuberculosis sequelae. Lancet Glob Health. (2021) 9:e1679–87. doi: 10.1016/S2214-109X(21)00367-3, PMID: 34798027 PMC8609280

[28] Brighton LJ Chilcot J Maddocks M. Social dimensions of chronic respiratory disease: stigma, isolation, and loneliness. Curr Opin Support Palliat Care. (2022) 16, 4:195–202. doi: 10.1097/SPC.0000000000000616 36102929

[29] Woo S Zhou W Larson JL. Stigma experiences in people with chronic obstructive pulmonary disease: an integrative review. Int J Chron Obstruct Pulmon Dis. (2021) 4:1647–59. doi: 10.2147/COPD.S306874 PMC8187000 34113096

[30] Nohra RG Morviller J-M Sacre H Salameh P Rothan-Tondeur M. Living with chronic obstructive pulmonary disease in Lebanon: a phenomenological study. East Mediterr Health J. (2022) 28:114–20. doi: 10.26719/emhj.22.027, PMID: 35304908

[31] Jerpseth H Knutsen IR Jensen KT Halvorsen K. Mirror of shame: patients experiences of late-stage COPD. A qualitative study. J Clin Nurs. (2021) 30:2854–62. doi: 10.1111/jocn.15792, PMID: 33934413

[32] Lundell S Wadell K Wiklund M Tistad M. Enhancing confidence and coping with stigma in an ambiguous interaction with primary care: a qualitative study of people with COPD. COPD. (2020) 17:53–542. doi: 10.1080/15412555.2020.1824217 32981381

[33] Simonÿ C Andersen IC Bodtger U Birkelund R. Breathing through a troubled life – a phenomenological-hermeneutic study of chronic obstructive pulmonary disease patients’ lived experiences during the course of pulmonary rehabilitation. Int J Qual Stud Health Well Being. (2019) 14:1647401. doi: 10.1080/17482631.2019.1647401, PMID: 31432771 PMC6713173

[34] Sigurgeirsdottir J Halldorsdottir S Arnardottir RH Gudmundsson G Bjornsson EH. COPD patients’ experiences, self-reported needs, and needs-driven strategies to cope with self-management. Int J Chron Obstruct Pulmon Dis. (2019) 14:1033–43. doi: 10.2147/COPD.S201068 31190788 PMC6529673

[35] Ribeiro RM Havik PJ Craveiro I. The circuits of healthcare: understanding healthcare seeking behaviour—a qualitative study with tuberculosis patients in Lisbon Portugal. PLoS One. (2019) 16:e0261688. doi: 10.1371/journal.pone.0261688 PMC8714083 34962944

[36] Marahatta SB Yadav RK Giri D Lama S Rijal KR Mishra SR. Barriers in the access, diagnosis and treatment completion for tuberculosis patients in central and western Nepal: a qualitative study among patients, community members and health care workers. PLoS One. (2020) 15:e0227293. doi: 10.1371/journal.pone.0227293, PMID: 31940375 PMC6961875

[37] Endalamaw A Gilks CF Ambaw F Chatfield MD Assefa Y. Satisfaction of tuberculosis patients to healthcare services at the global level: a systematic review. Health Soc Care Community. (2022) 30:e3435–46. doi: 10.1111/hsc.13953, PMID: 35920598 PMC10087702

[38] Romanowski K Cook VJ Gilbert M Johnston JC. Using a theory-informed approach to guide the initial development of a post-tuberculosis care package in British Columbia Canada. BMC Health Serv Res. (2023) 23:805. doi: 10.1186/s12913-023-09835-4, PMID: 37501183 PMC10375626

[39] WHO. Policy brief on tuberculosis-associated disability. (2023). Available at: https://iris.who.int/bitstream/handle/10665/373679/9789240077799-eng.pdf?sequence=1 (accessed February 28, 2024)

[40] Jones R Muyinda H Nyakoojo G Kirenga B Katagira W Pooler J. Does pulmonary rehabilitation alter patients' experiences of living with chronic respiratory disease? A qualitative study. Int J Chron Obstruct Pulmon Dis. (2018) 8:2375–85. doi: 10.2147/COPD.S165623 PMC6087019 30122917

[41] Mademilov M Mirzalieva G Yusuf ZK Orme MW Bourne C Akylbekov A. What should pulmonary rehabilitation look like for people living with post-tuberculosis lung disease in the Bishkek and Chui region of the Kyrgyz Republic? A qualitative exploration. BMJ Open. (2022) 12:e053085. doi: 10.1136/bmjopen-2021-053085, PMID: 35121602 PMC8819799

