# Supplementary material for: “You find yourself in a very humiliating situation”: experiences of people living with post-tuberculosis lung disease in Brazil
Source: Front Public Health. 2024 Dec 24;12:1431881. doi: 10.3389/fpubh.2024.1431881 (PMC11703962; doi:10.3389/fpubh.2024.1431881)
Supplement: Supplementary file 1 [file Table_1.DOCX]

Interview Guide for Patients and Providers

**Questions and probes for patients**

01- How would you describe your day so far, since waking up?

02- How is your health currently?

03- What is it like living with post-TB lung disease (TB sequelae)?

04- What changed in your life after you received the post-TB lung disease diagnosis?

- Could you tell me more about this aspect? (probe)

05- How did you feel after you were diagnosed with post-tuberculosis lung disease (TB sequelae)?

06- How did you feel when you found out about the TB sequelae even after the treatment?

- What was it like talking about the diagnosis to the people closest to you? (probe)

- What were their reactions? (probe)

07- How is it for you to access healthcare facilities nowadays?

- How would you describe possible barriers and facilitators in accessing health services? (probe)

08- What kind of response from healthcare workers do you usually receive when you go to a health care facility because of post-TB lung disease?

09- What kind of limitations, if any, do you face in your daily life activities as a result of post-tuberculosis lung disease (TB sequelae)?

- How do you deal with these limitations? (probe)

- How do these limitations impact your life? (probe)

10- What do you do to lessen these limitations?

- How do you feel after you have received this treatment? (probe)

11- Is there anything you would like to add that you feel is important to mention?

12- Is there anything else you would like to share with me?
