# Supplementary material for: “You find yourself in a very humiliating situation”: experiences of people living with post-tuberculosis lung disease in Brazil
Source: Front Public Health. 2024 Dec 24;12:1431881. doi: 10.3389/fpubh.2024.1431881 (PMC11703962; doi:10.3389/fpubh.2024.1431881)
Supplement: Supplementary file 2 [file Table_2.DOCX]

**Questions and probes for providers**

01- How would you describe your workday so far?

02- What is the burden you observe among patients with PTLD?

- Could you tell me a little more about how these patients demand of you as a health professional?

03- How has it been for you to work with people living with PTLD?

04- How do you imagine these people feel at the time of diagnosis?

- For you, what are their main fears and concerns at that moment? (probe)

05- How does the life of people living with PTLD change after the diagnosis?

- What difficulties do you perceive in their lives? (probe)

06- How do you perceive their access to healthcare services?

- Could you tell me a little more about how they are received by the health team?

07- How do health professionals typically respond when people with PTLD seek health care services?

- Is there any kind of reaction that impacts the willingness of these individuals to return to healthcare facilities? (probe)

- Could you detail this experience a little more? (probe)

08- How do you perceive possible limitations in the lives of people living with PTLD?

- Could you talk more about how stigma might be a limiting factor for them? (probe)

09- What do you think they could or should do to reduce the impact of these limitations?

10- Is there anything you would like to add that you think is important to mention?

11- Is there anything else you would like to share with me?
